# The impact of intraoperative hypotension on postoperative acute kidney injury, mortality and length of stay following off-pump coronary artery bypass grafting surgery: a single-center retrospective cohort study

**DOI:** 10.1186/s12871-024-02616-4

**Published:** 2024-07-05

**Authors:** Cheng Xiao, Ming Yang, Lei Cao, Fang Chen, Sheng Jing, Yuting Tan, Hong Li

**Affiliations:** Department of Anesthesiology, Second Affiliated Hospital of Army Medical University, PLA, No. 83 Xinqiao Road, Chongqing, 400037 China

**Keywords:** Off-pump coronary artery bypass grafting, Intraoperative hypotension, Acute kidney injury, Mortality, Length of stay

## Abstract

**Background:**

Off-pump coronary artery bypass grafting (OPCABG) presents distinct hemodynamic characteristics, yet the relationship between intraoperative hypotension and short-term adverse outcomes remains clear. Our study aims to investigate association between intraoperative hypotension and postoperative acute kidney injury (AKI), mortality and length of stay in OPCABG patients.

**Methods:**

Retrospective data of 494 patients underwent OPCABG from January 2016 to July 2023 were collected. We analyzed the relationship between intraoperative various hypotension absolute values (MAP > 75, 65 < MAP ≤ 75, 55 < MAP ≤ 65, MAP ≤ 55 mmHg) and postoperative AKI, mortality and length of stay. Logistic regression assessed the impacts of exposure variable on AKI and postoperative mortality. Linear regression was used to analyze risk factors on the length of intensive care unit stay (ICU) and hospital stay.

**Results:**

The incidence of AKI was 31.8%, with in-hospital and 30-day mortality at 2.8% and 3.5%, respectively. Maintaining a MAP greater than or equal 65 mmHg [odds ratio (OR) 0.408; *p* = 0.008] and 75 mmHg (OR 0.479; *p* = 0.024) was significantly associated with a decrease risk of AKI compared to MAP less than 55 mmHg for at least 10 min. Prolonged hospital stays were linked to low MAP, while in-hospital mortality and 30-day mortality were not linked to IOH but exhibited correlation with a history of myocardial infarction. AKI showed correlation with length of ICU stay.

**Conclusions:**

MAP > 65 mmHg emerges as a significant independent protective factor for AKI in OPCABG and IOH is related to length of hospital stay. Proactive intervention targeting intraoperative hypotension may provide a potential opportunity to reduce postoperative renal injury and hospital stay.

**Trial registration:**

ChiCTR2400082518. Registered 31 March 2024. https://www.chictr.org.cn/bin/project/edit?pid=225349.

## Background

Coronary artery disease remains a leading cause of death worldwide [1]. Off-pump coronary artery bypass grafting (OPCABG), as a myocardial revascularization technique for treating this disease [2], offers advantages in maintaining autonomous blood flow of coronary artery, reducing myocardial ischemia/reperfusion injury, and avoiding non-physiological perfusion and cardiac arrest during cardiopulmonary bypass. Acute kidney injury (AKI) is a common complication that is associated with prolonged hospital stay, higher mortality rates and increased healthcare costs following both cardiac and major non-cardiac surgeries [3, 4]. Literature suggests a correlation between intraoperative hypotension (IOH) and various postoperative complications such as AKI [5], myocardial injury [6], stroke [7], delirium [8] and mortality [9] among patients undergoing non-cardiac surgery. IOH may also be a variable risk factor for major adverse events in patients undergoing cardiac surgery [10]. Accordingly, there’s a growing focus on targeting blood pressure management during the perioperative period of both non-cardiac surgery and cardiac surgery to prevent postoperative AKI and adverse outcomes. In patients undergoing non-cardiac surgery, maintaining an average arterial pressure (MAP) of 65 mmHg is suggested as the management threshold [11], but research on OPCABG is scarce. However, OPCABG patients constitute a distinct population compared to the heterogeneous group undergoing ‘cardiac surgery’ due to the absence of cardiopulmonary bypass. In addition, OPCABG has its unique hemodynamic characteristics, the heart and the major vessels need to be moved and squeezed many times during the operation, significantly impacting hemodynamic stability. Moreover, severe hemodynamic fluctuations are frequent during specific time periods such as anesthesia induction and fixed coronary artery compression, heightening the likelihood of IOH and complicating anesthesia management. Surgical-induced circulatory fluctuations and low cardiac output syndrome may contribute to organ hypoperfusion. Nonetheless, there remains a lack of consensus regarding the ideal blood pressure intervention threshold specific to OPCABG, and the impact of IOH on early postoperative renal function following OPCABG requires further investigation.

The authors, therefore, decided to retrospectively investigate the association between IOH and postoperative acute kidney injury (AKI), mortality and length of stay in OPCABG patients.

## Methods

### Study design and setting

The author’s local ethics committee approved this single-center retrospective observational study, and granted a waiver of informed consent. The study included patients who underwent OPCABG from January 2016 to July 2023 at the department of anesthesiology, second affiliated hospital of army medical university, PLA. The exclusion criteria included (1) patients undergoing dialysis, (2) unavailability of a baseline renal function test, (3) previous nephrectomy or renal transplantation, (4) minimally invasive CABG, (5) OPCABG combined with other surgical procedures, (6) re-operation. This study procedures were followed in accordance with the ethical standards of the responsible committee on human experimentation and with the Helsinki Declaration of 1975.

Data were collected from the hospital’s Electronic Patient Record (EPR) System and electronic anesthesia record-keeping system, which are both standard documentation tools in our institute for recording patient care details.

### Data collection

Demographic and perioperative factors associated with postoperative AKI, which were the primary outcome in previous studies, were recorded, including sex, age, body mass index (BMI), smoking status, systemic immune-inflammation index (SII), preoperative serum creatinine (sCr), left ventricular ejection fraction (LVEF), comorbidities (diabetes, hypertension, prior myocardial infarction (MI), percutaneous coronary intervention (PCI), stroke, chronic kidney disease (CKD), dyslipidemia, hypoproteinemia), intraoperative fluid therapy (crystalloid, colloidal solution, autologous blood, red blood cells, plasma), bleeding, operation time, in-hospital mortality, 30-day mortality, length of ICU stay and LOS.

During surgery, all patients underwent invasive arterial blood pressure monitoring. Blood pressure were recorded before and after the induction of anesthesia and at 5-minute intervals until the patient’s departure from the operating theater. Raw MAP values were extracted from the anesthesia records and filtered for obvious artifacts, excluding values < 0 or > 250mmHg. This study analyzed four absolute MAP thresholds (MAP > 75, 65 < MAP ≤ 75, 55 < MAP ≤ 65, MAP ≤ 55 mmHg) after assessing relevant literature, IOH was defined by at least one MAP measurement below the threshold for at least 10 min.

Midazolam, etomidate, sufentanil, and rocuronium were administered for anesthesia induction, ensuring a slow and stable induction process. In cases of low blood pressure during the operation, pressor medications such as dopamine (3-10ug/kg/min) and/or norepinephrine (0.1-0.25ug/kg/min) can be administered. If significant fluctuations in heart rate and blood pressure occur during cardiac manipulation, the surgeon should give priority to flatten the heart and suspend the procedure temporarily. Trendelenburg positioning and fluid rehydration should be initiated, with intravenous administration of norepinephrine at a dosage of 4-8ug if necessary. The management of blood pressure and interventions to address hypotension were at the discretion of the treating anesthesiologist.

### Variable definitions

The primary outcome measure, postoperative AKI, was defined based on the KDIGO guidelines, utilizing either a 0.3 mg/dL increase in creatinine level within the first 48 h after surgery or a 1.5-fold increase in the first 7 days after surgery. In this retrospective study, we exclusively relied on elevated creatinine level as the diagnostic criterion of AKI. Preoperative sCr was defined as the sCr obtained closest to the date of surgery. Peak postoperative sCr was defined as the highest creatinine level recorded within the 7 days following surgery.

### Statistical analysis

Statistical analysis was conducted using IBM SPSS Version 26.0 (SPSS Inc., Chicago, IL, USA). Continuous variables were presented as median and interquartile range (IQR), while categorical variables were expressed as absolute values and/or percentages. Between-group differences for quantitative variables were assessed using the Mann-Whitney U-test, while categorical variables were analyzed using the chi-square test. A significance level of *p* < 0.05 (two-tailed) was regarded statistically significant. Variables with *p* values < 0.05 from univariate analysis or clinically correlated with AKI and mortality were included in multivariable logistic regression model. For AKI, the covariates included preoperative sCr, hypertension, preoperative CKD, colloidal solution, red blood cells, plasma, and MAP. For in-hospital mortality, the covariates included prior MI, postoperative AKI, and MAP. For 30-day mortality, the covariates included smoker status, prior MI, postoperative AKI, and MAP. Length of ICU stay and LOS were evaluated using a linear regression model.

## Results

**Fig. 1:**
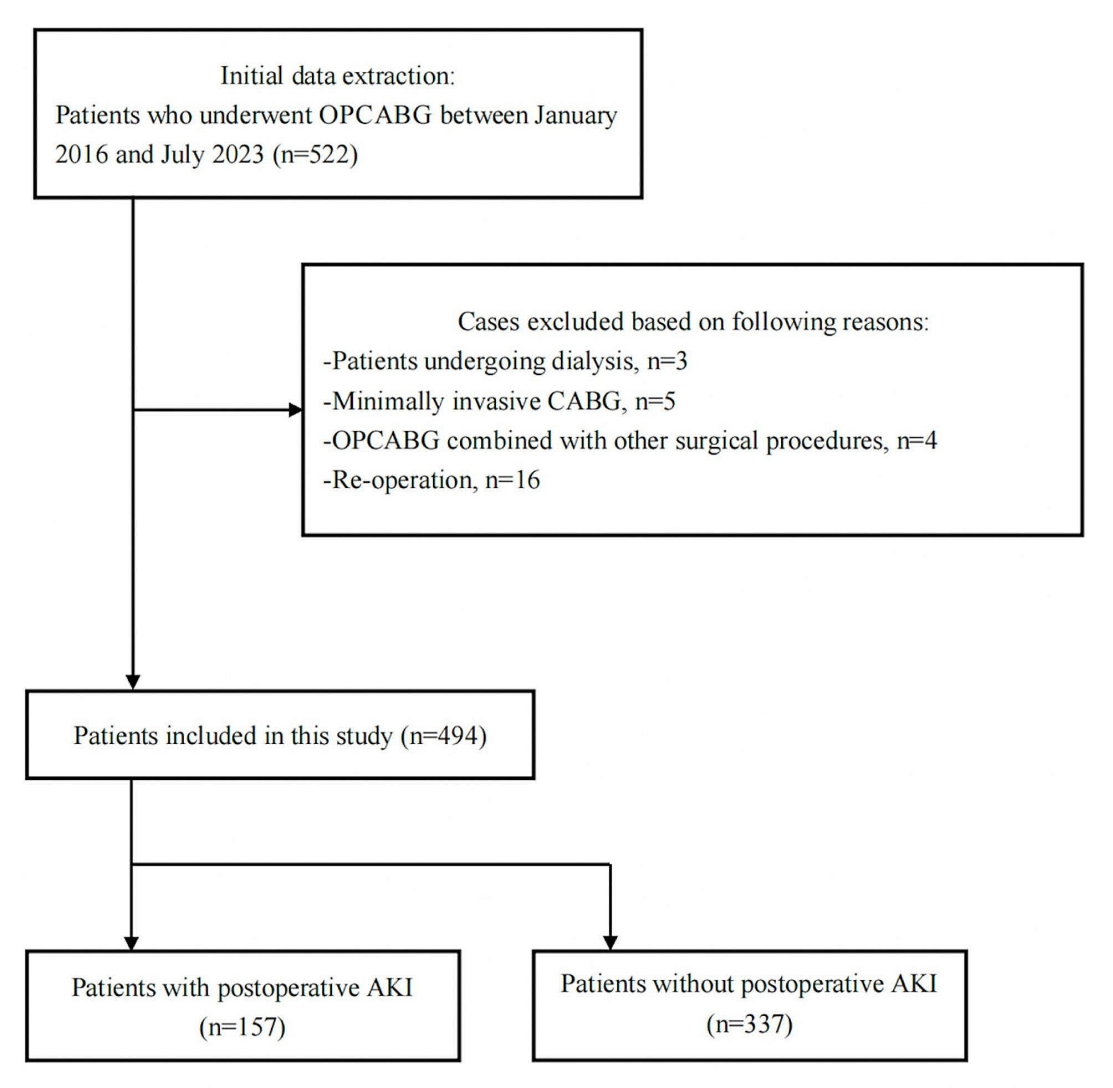
Flow chart of the study. OPCABG, off-pump coronary artery bypass grafting; eGFR, estimated glomerular filtration rate; CABG, coronary artery bypass grafting; AKI, acute kidney injury

A total of 522 patients underwent OPCABG between January 2016 and July 2023, of whom 494 patients met our inclusion and exclusion criteria (Fig. 1). 157 patients (31.8%) were diagnosed with postoperative AKI. The distribution of AKI stages, according to the KDIGO classification, was as follows: stage 1, 127 (25.7%) patients, stage 2, 23 (4.7%) patients, and stage 3, 7 (1.4%) patients. The perioperative characteristics of patients with and without AKI were summarized in Table 1. Compared to the non-AKI group, statistically significant differences were observed in the preoperative sCr levels, history of hypertension and CKD, intraoperative colloid infusion, as well as the transfusion of red blood cells and plasma in AKI group.

**Table 1:** Patient-related factors Associated with postoperative AKI.

	Total (*n* = 494)	No AKI (*n* = 337)	AKI (*n* = 157)	Statistic	*P*
Age, yr	63.00 (55.00, 68.00)	62.00 (55.00, 67.00)	64.00 (56.00, 69.00)	Z=-1.96	0.050
Female, n (%)	100 (20.24)	69 (20.47)	31 (19.75)	χ²=0.04	0.851
BMI, n (%)				χ²=2.30	0.129
< 24 kg/m^2^	188 (38.45)	136 (40.72)	52 (33.55)		
≥ 24 kg/m^2^	301 (61.55)	198 (59.28)	103 (66.45)		
Smoker, n (%)	152 (30.83)	102 (30.36)	50 (31.85)	χ²=0.11	0.739
SII	471.81 (321.42, 661.09)	474.17 (322.59, 657.43)	466.27 (320.72, 661.29)	Z=-0.27	0.788
preoperative sCr, µmol/L	78.70 (67.20, 93.07)	76.70 (67.00, 88.30)	86.30 (70.10, 102.30)	Z=-4.08	< 0.001*
LVEF	62.00 (58.00, 66.93)	63.00 (58.00, 67.00)	62.00 (58.00, 66.60)	Z=-0.05	0.958
Previous medical history, n (%)					
Diabetes, n (%)	174 (35.22)	110 (32.64)	64 (40.76)	χ²=3.10	0.078
Hypertension, n (%)	292 (59.11)	183 (54.30)	109 (69.43)	χ²=10.14	0.001*
Prior MI, n (%)	113 (22.92)	80 (23.74)	33 (21.15)	χ²=0.40	0.525
PCI, n (%)	83 (16.80)	56 (16.62)	27 (17.20)	χ²=0.03	0.872
Stroke, n (%)	48 (9.72)	28 (8.31)	20 (12.74)	χ²=2.40	0.122
CKD, n (%)	13 (2.63)	5 (1.48)	8 (5.10)	χ²=4.13	0.042*
Dyslipidaemia, n (%)	211 (43.78)	140 (42.55)	71 (46.41)	χ²=0.63	0.428
Hypoproteinemia, n (%)	44 (8.94)	29 (8.66)	15 (9.55)	χ²=0.11	0.745
Intraoperative					
Crystalloid, ml	1400.00 (1200.00, 1750.00)	1415.00 (1200.00, 1750.00)	1400.00 (1150.00, 1800.00)	Z=-0.85	0.396
Colloidal solution, ml	205.00 (0.00, 500.00)	100.00 (0.00, 500.00)	400.00 (0.00, 700.00)	Z=-3.31	< 0.001*
Autologous blood, ml	290.50 (179.25, 450.00)	285.00 (180.00, 442.00)	310.00 (179.00, 468.00)	Z=-1.23	0.218
Red blood cells, ml	0.00 (0.00, 200.00)	0.00 (0.00, 0.00)	0.00 (0.00, 400.00)	Z=-2.14	0.032*
Plasma, ml	0.00 (0.00, 400.00)	0.00 (0.00, 400.00)	0.00 (0.00, 400.00)	Z=-2.04	0.041*
Bleeding, ml	350.00 (300.00, 450.00)	350.00 (300.00, 430.00)	350.00 (300.00, 500.00)	Z=-1.52	0.130
Operation time, min	260.00 (230.00, 300.00)	260.00 (230.00, 300.00)	260.00 (225.00, 300.00)	Z=-0.71	0.475

BMI, body mass index; SII, systemic immune-inflammation index; sCr, serum creatinine; LVEF, left ventricular ejection fraction; MI, myocardial infarction; PCI, percutaneous coronary intervention; CKD, chronic kidney disease

* Statistically significant

The in-hospital mortality and 30-day mortality rates were 2.8% and 3.5%, respectively. Regarding in-hospital mortality, no statistical differences were observed among all baseline characteristics between the 2 groups, except for the history of MI. Both smoking and prior MI were found to be statistically significant factors for postoperative 30-day mortality (Table 2).

**Table 2:** Patient-related factors Associated with In-hospital mortality and 30-day mortality

	Total (*n* = 494)	In-hospital mortality		Statistic	*P*	Total (*n* = 490)	30-day mortality		Statistic	*P*
		No (*n* = 480)	Yes (*n* = 14)				No (*n* = 473)	Yes (*n* = 17)		
Age, yr	63.00 (55.00, 68.00)	63.00 (55.00, 68.00)	66.00 (65.00, 70.00)	Z=-1.93	0.053	63.00 (55.00,68.00)	63.00 (55.00, 68.00)	66.00 (58.00, 70.00)	Z=-1.63	0.103
Female, n (%)	100 (20.24)	97 (20.21)	3 (21.43)	χ²=0.00	1.000	99 (20.20)	96 (20.30)	3 (17.65)	χ²=0.00	1.000
BMI, n (%)				χ²=0.81	0.367				χ²=3.12	0.077
< 24 kg/m^2^	188 (38.45)	181 (38.11)	7 (50.00)			186 (38.35)	176 (37.61)	10 (58.82)		
≥ 24 kg/m^2^	301 (61.55)	294 (61.89)	7 (50.00)			299 (61.65)	292 (62.39)	7 (41.18)		
Smoker, n (%)	152 (30.83)	145 (30.27)	7 (50.00)	χ²=1.64	0.200	152 (31.08)	143 (30.30)	9 (52.94)	χ²=3.93	0.047*
Skii,	471.81 (321.42, 661.09)	471.81 (322.10, 661.24)	493.75 (328.42, 561.86)	Z=-0.16	0.872	471.16 (321.02, 659.88)	470.57 (321.01, 661.22)	537.74 (379.02, 620.45)	Z=-0.37	0.710
Preoperative sCr, µmol/L	78.70 (67.20, 93.07)	78.70 (67.07, 92.85)	78.10 (72.92, 101.65)	Z=-0.49	0.621	78.65 (67.12, 93.07)	78.60 (66.80, 92.70)	81.20 (73.30, 103.40)	Z=-1.04	0.299
LVEF	62.00 (58.00, 66.93)	62.25 (58.00, 66.80)	62.00 (58.52, 66.75)	Z=-0.01	0.992	62.00 (58.00, 67.00)	62.60 (58.00, 67.00)	60.50 (53.00, 66.00)	Z=-1.03	0.301
Previous medical history, n (%)										
Diabetes, n (%)	174 (35.22)	170 (35.42)	4 (28.57)	χ²=0.06	0.807	173 (35.31)	168 (35.52)	5 (29.41)	χ²=0.27	0.605
Hypertension, n (%)	292 (59.11)	281 (58.54)	11 (78.57)	χ²=2.26	0.133	289 (58.98)	276 (58.35)	13 (76.47)	χ²=2.23	0.136
Prior MI, n (%)	113 (22.92)	105 (21.92)	8 (57.14)	χ²=7.66	0.006*	113 (23.11)	103 (21.82)	10 (58.82)	χ²=10.65	0.001*
PCI, n (%)	83 (16.80)	81 (16.88)	2 (14.29)	χ²=0.00	1.000	82 (16.73)	80 (16.91)	2 (11.76)	χ²=0.05	0.820
Stroke, n (%)	48 (9.72)	47 (9.79)	1 (7.14)	χ²=0.00	1.000	47 (9.59)	45 (9.51)	2 (11.76)	χ²=0.00	1.000
CKD, n (%)	13 (2.63)	12 (2.49)	1 (7.14)	-	0.315	13 (2.65)	12 (2.54)	1 (5.88)	-	0.372
Dyslipidaemia, n (%)	211 (43.78)	204 (43.50)	7 (53.85)	χ²=0.55	0.458	209 (43.72)	201 (43.51)	8 (50.00)	χ²=0.27	0.607
Hypoproteinemia, n (%)	44 (8.94)	43 (9.00)	1 (7.14)	χ²=0.00	1.000	44 (9.02)	43 (9.13)	1 (5.88)	χ²=0.00	0.977
Intraoperative										
Crystalloid, ml	1400.00 (1200.00, 1750.00)	1400.00 (1200.00, 1750.00)	1535.00 (1262.50, 1780.00)	Z=-0.69	0.489	1400.00 (1200.00, 1750.00)	1400.00 (1200.00, 1750.00)	1430.00 (1250.00, 1720.00)	Z=-0.26	0.793
Colloidal solution, ml	205.00 (0.00, 500.00)	200.00 (0.00, 500.00)	400.00 (50.00, 1000.00)	Z=-1.39	0.164	230.00 (0.00, 500.00)	210.00 (0.00, 500.00)	300.00 (0.00, 1000.00)	Z=-0.72	0.474
Autologous blood, ml	290.50 (179.25, 450.00)	291.50 (180.00, 448.50)	188.50 (162.25, 486.00)	Z=-0.64	0.521	290.50 (179.25, 450.00)	291.00 (180.00, 450.00)	220.00 (169.00, 450.00)	Z=-0.57	0.567
Red blood cells, ml	0.00 (0.00, 200.00)	0.00 (0.00, 200.00)	0.00 (0.00, 0.00)	Z=-0.94	0.347	0.00 (0.00, 200.00)	0.00 (0.00, 200.00)	0.00 (0.00, 0.00)	Z=-0.76	0.450
Plasma, ml	0.00 (0.00, 400.00)	0.00 (0.00, 400.00)	0.00 (0.00, 400.00)	Z=-0.38	0.706	0.00 (0.00, 400.00)	0.00 (0.00, 400.00)	0.00 (0.00, 400.00)	Z=-0.46	0.643
Bleeding, ml	350.00 (300.00, 450.00)	350.00 (300.00, 450.00)	350.00 (300.00, 437.50)	Z=-0.14	0.889	350.00 (300.00, 450.00)	350.00 (300.00, 450.00)	350.00 (300.00, 450.00)	Z=-0.06	0.949
Operation time, min	260.00 (230.00, 300.00)	260.00 (230.00, 300.00)	237.50 (225.00, 295.00)	Z=-0.95	0.340	260.00 (230.00, 300.00)	260.00 (230.00, 300.00)	260.00 (225.00, 300.00)	Z=-0.59	0.553

BMI, body mass index; SII, systemic immune-inflammation index; sCr, serum creatinine; LVEF, left ventricular ejection fraction; MI, myocardial infarction; PCI, percutaneous coronary intervention; CKD, chronic kidney disease

* Statistically significant

In the univariate analysis, maintaining a MAP greater than or equal 65 mmHg [odds ratio (OR) 0.375; *p* = 0.002] and 75 mmHg (OR 0.419; *p* = 0.005) was significantly associated with a decrease risk of AKI compared to MAP less than 55 mmHg for at least 10 min. After performing multivariable analysis and adjusting for confounders, it was observed that compared to MAP less than 55 mmHg, a MAP threshold greater than or equal to 65 mmHg (OR 0.408; *p* = 0.008) and 75 mmHg (OR 0.479; *p* = 0.024) were helpful to reduce the occurrence of AKI (Table 3). Preoperative sCr, and CKD, intraoperative transfusion of red blood cells and plasma did not demonstrate statistical significance. Notably, a history of hypertension (OR 1.879; *p* = 0.003) and intraoperative colloid infusion (OR 1.001; *p* = 0.004) emerged as risk factors for OPCABG (Table 3).

**Table 3:** Multivariable models for postoperative AKI, In-hospital mortality, postoperative 30-day mortality, length of ICU stay and LOS.

		95% CI	*P* value
**AKI**	**Adjusted Odds Ratio (OR)**		
Preoperative sCr, µmol/L	1.005	0.999, 1.011	0.091
Hypertension, n (%)	1.879	1.237, 2.855	0.003*
CKD, n (%)	2.971	0.844, 10.455	0.090
Colloidal solution, ml	1.001	1.000, 1.001	0.004*
Red blood cells, ml	1.000	0.999, 1.001	0.671
Plasma, ml	1.000	0.999, 1.001	0.758
MAP ≦ 55		Ref	0.058
55 < MAP ≦ 65, mmHg	0.738	0.178, 3.065	0.676
65 < MAP ≦ 75, mmHg	0.408	0.210, 0.791	0.008*
MAP>75, mmHg	0.479	0.252, 0.908	0.024*
**In-hospital mortality**	**Adjusted Odds Ratio (OR)**		
Prior MI, n (%)	5.139	1.726, 15.301	0.003*
AKI, n (%)	2.551	0.858, 7.588	0.092
MAP ≦ 55		Ref	0.897
55 < MAP ≦ 65, mmHg	0.000	0.000	0.999
65 < MAP ≦ 75, mmHg	2.336	0.264, 20.656	0.445
MAP>75,mmHg	1.936	0.225, 16.628	0.547
**30-day mortality**	**Adjusted Odds Ratio (OR)**		
Smoker, n (%)	2.035	0.734, 5.462	0.172
Prior MI, n (%)	4.949	1.796, 13.637	0.002*
AKI, n (%)	2.690	0.983, 7.364	0.054
MAP ≦ 55		Ref	0.991
55 < MAP ≦ 65, mmHg	0.000	0.000	0.999
65 < MAP ≦ 75, mmHg	1.132	0.221, 6.082	0.885
MAP>75,mmHg	0.947	0.184, 4.881	0.948
**Length of ICU stay**	**Coefficient**		
LVEF	-0.052	-0.083, -0.022	0.001*
Colloidal solution, ml	0.001	0.000, 0.001	0.024*
AKI	1.542	0.970, 2.114	< 0.001*
**LOS**	**Coefficient**		
PCI, n (%)	3.435	1.103, 5.767	0.004*
Bleeding, ml	0.006	0.002, 0.010	0.015*
MAP, mmHg	0.005	0.001, 0.009	0.010*

Multivariable analysis showed a significant association between in-hospital mortality and postoperative 30-day mortality with prior MI, exhibiting OR 5.139 (*p* = 0.003) and OR 4.949 (*p* = 0.002) respectively. Prior MI was identified as the sole significant predictor of both in-hospital mortality and 30-day mortality. MAP showed no statistical difference in either in-hospital mortality or postoperative 30-day mortality.

Length of ICU stay and LOS outcomes were analyzed using a stepwise linear regression model. AKI was associated with 1.542 (95% CI: 0.970–2.114, *p* < 0.001) days prolonged stay in ICU than no AKI. Patients with lower LVEF and greater colloidal solution infusion during the operation were both related to the prolonged stays in the ICU. Preoperative PCI and greater intraoperative blood loss were identified as risk factors for extended in-hospital stays. For every threshold increase in MAP (MAP > 75, 65 < MAP ≤ 75, 55 < MAP ≤ 65, MAP ≤ 55 mmHg), the LOS decreased by 1.542 days (95% CI: 0.472–2.991, *p* = 0.007).

## Discussion

The study evaluated the relationship between various levels of intraoperative hypotension and postoperative AKI, as well as significant adverse outcomes during OPCABG. Its objective was to identify improved intraoperative management strategies to prevent kidney injury and enhance clinical outcomes, potentially benefiting the OPCABG population. Within our cohort of 494 patients, 157 (31.8%) experienced AKI following the first isolated OPCABG, consistent with findings from previous studies [12]. We found that maintaining the MAP threshold ≥ 65 mmHg for at least 10 min was significantly associated with a decrease risk of AKI. Furthermore, for every decrease in MAP threshold (MAP > 75, 65 < MAP ≤ 75, 55 < MAP ≤ 65, MAP ≤ 55 mmHg), LOS increased by 1.542 days. However, we found no direct correlation between MAP and in-hospital mortality, 30-days mortality, or length of ICU stay.

International consensus statements strongly support the association between IOH and postoperative adverse events including MI, AKI, mortality. They particularly emphasis that AKI is a function of hypotension severity and duration [13]. Extensive analysis of multiple large-scale observational studies across diverse surgical populations had identified MAP < 60 ~ 70 mmHg as critical thresholds where significant adverse events occur, particularly evident in non-cardiac surgeries. Our findings align with the consensus and prior research [10, 14], which emphasized the increased risk of postoperative renal injury associated with MAP < 65 mmHg during the CPB phase of cardiac surgery. From a physiological standpoint, the suitability of a universal blood pressure threshold for various surgery types is questionable, as the relationship between IOH and organ damage varies among individuals and surgical procedures. Therefore, formulating tailored intraoperative blood pressure management for distinct surgical types seems more promising in preventing organ dysfunction than relying on a single universal blood pressure threshold. Our focus was on OPCABG, a procedure known for its hemodynamic instability during surgery. Although falling within the realm of cardiac surgery, OPCABG does not involve cardiopulmonary bypass. Our aim was to explore optimal intraoperative blood pressure management for such patients and propose validated blood pressure targets for prospective trials investigating individualized hypotension management to reduce adverse outcomes, which holds promise as a potential practice standard. Equally important, the absence of such date brings challenges to clinicians in guiding the daily management of these patients. Based on our findings, we recommend adopting MAP ≥ 65 mmHg as the absolute threshold, as it enables the identification of more patients experiencing hypotension and has been associated with reduction postoperative AKI occurrence in our study. Although we offer possible explanations for our data, it is crucial to recognize the intrinsic limitations of our single-center study. The sample size restricts the generalizability of our findings to wider populations. While our results align with existing consensus and previous research, the limited external validity of single-center studies implies the necessity for validation in larger, multicenter cohorts to ensure broader relevance. Additionally, we observed that, apart from the previously recognized history of hypertension [15–17], intraoperative colloid infusion emerged as closely associated with AKI, a factor that has been less emphasized in classical prediction model. At our center, two colloidal solutions, succinyl gelatin and hydroxyethyl starch, were utilized, with the former being more commonly employed than the latter. Numerous studies have confirmed the nephrotoxicity of hydroxyethyl starch [18, 19]. Jeong [20] also emphasized the need for cautious intraoperative use of hydroxyethyl starch during OPCABG. However, evidence regarding the benefits and harms of succinyl gelatin remains limited. Smart L [21] found that fluid resuscitation after heart surgery with succinyl gelatin (4%) was related to the increase of biomarker concentration of renal tubular injury and dysfunction compared with crystalloid fluid. Katona [22] similarly reported a significant association between gelatin administration and an increased risk for early postoperative AKI in a dose-dependent manner. These findings raise concern regarding the potential for intravenous gelatin solution to contribute to clinically relevant postoperative AKI. Given the retrospective nature of our study, we were unable to analyze succinyl gelatin and hydroxyethyl starch separately to obtain more precise evidence. Nevertheless, our research suggested that limiting or avoiding colloid administration during OPGAB might be an effective intervention.

The relationship between IOH and mortality has yielded conflicting results in previous studies. De la Hoz [10] identified a statistically significant association between each 10-minute exposure to IOH and composite outcomes including postoperative MI, AKI and death. Similarly, Nakanishi T and Cai JH [23, 24] reported correlation between IOH and postoperative AKI and mortality in retrospective study and meta-analysis. Esther M [25] highlighted a graded association between IOH and postoperative MI, AKI and mortality, suggesting that the depth of hypotension carries more weight than its duration. However, contrary to these findings, our study found no correlation between IOH and in-hospital mortality or 30-days mortality. One possible explanation for these discrepancies is that the referenced studies primarily focused on non-cardiac surgeries or cardiac surgeries involving cardiopulmonary bypass. Differences in patient populations, such as variations in baseline health conditions and risk factors, could significantly influence the outcomes. Our observations are consistent with those of several other studies [26–29], which also concluded that IOH may not be a primary determinant of postoperative mortality. For instance, Kluger evaluated the relationship between 3-, 30- and 365-day mortality and intraoperative hypotension absolute values (MAP ≤ 55, 60, 65, 70 and 75 mmHg) and found that some univariate associations between hypotension and mortality. However, these associations were not evident in multivariable analysis. D’Amico compared intraoperative blood pressure management aiming for MAP ≤ 60 mmHg with targeted MAP > 60 mmHg in an observational study. The primary outcome, all-cause mortality at the longest available follow-up, as pooled form randomized evidence, showed no association with a target intraoperative MAP ≤ 60 mmHg. Nevertheless, further research, particularly multicenter studies with larger cohorts and standardized definitions, is needed to resolve these inconsistencies and validate our findings.

Our study also investigated the relationship between IOH exposure and length of ICU stay and hospitalization time, which are key performance indicators of hospital management and health care system efficiency, especially post the full implementation of the Diagnosis-Related Group (DRG) system in China. At the levels observed in this study, we were unable to demonstrate a relationship between intraoperative hypotension and length of ICU stay. The latter was related to colloid infusion and AKI, potentially due to the increased likelihood of patients with AKI requiring intensive care and renal replacement therapy [30]. Our results suggested that the LOS would be reduced by 1.731 days for every threshold increase in MAP during operation. This may be related to inadequate blood perfusion of whole body tissues and organs caused by hypotension, as well as reperfusion injury following hypotension correction, which may be one among the primary mechanisms of organ tissue injury, especially in organs with high oxygen consumption rates per unit time and sensitivity to ischemia and hypoxia [31]. Zhang [32] found that intraoperative diastolic hypotension might increase the incidence of postoperative complications and prolong postoperative hospitalization. In addition, they found that hypotension could heighten the risk of postoperative infections, which was related to organ perfusion and minimum tissue oxygen saturation. Vassilios [33] confirmed that persistent hypotension could lead to a higher incidence of complications, especially cardiovascular, pulmonary and gastrointestinal complications, ultimately prolong hospitalization time. Prolonged hospitalization time exacerbates financial burdens, resulting in various social issues and potential doctor-patient tensions. Therefore, correcting intraoperative hypotension presents a potential opportunity to reduce hospitalization time and related expenses [34].

### Limitations

We acknowledge several limitations of our study. First, our study is a single-center observational analysis, limited by its retrospective nature, precluding the establishment of causal influences and susceptible to the influence of unpredictable factors. Although we attempted to mitigate selection bias by multivariate statistical methods, complete elimination of residual confounding remains challenging. Given the absence of a comparable prospective database on the relationship between IOH and postoperative AKI, mortality and LOS during OPCABG, our study serves as a compensatory study. Secondly, in the univariate analysis, the baseline level of preoperative sCr and CKD history between the no-AKI and AKI group were unbalanced, potentially affecting the occurrence rate of intraoperative hypotension. However, the median and interquartile range of baseline sCr in both groups fell within the normal range. Subsequent multivariate analysis showed no significant differences in sCr and CKD. This suggests that the impact of baseline imbalance on the relationship between IOH and AKI is relatively minor. Thirdly, our study did not account for hypotension occurrences beyond the intraoperative stages due to limitations in measurement and recording frequency. While the short-term but highly monitored intraoperative stage was evaluated, long-term postoperative hypotension may be more closely related to adverse outcomes. Furthermore, we didn’t collect the time-weighted average of each threshold, precluding a thorough analysis of the influence of the duration and severity of IOH on AKI, mortality and LOS outcomes. Nevertheless, our results are consistent with the international consensus statement on non-cardiac surgery, validating previous findings of a specific correlation between IOH and AKI, mortality and LOS. Our study underscores the importance of intraoperative hemodynamic management. Moving forward, researchers may focus on refining risk prediction models and developing targeted interventions aimed at mitigating IOH-related complications. Additionally, prospective multicenter studies involving larger cohorts are warranted to validate and extend our observations.

## Conclusion

In conclusion, our study in patients undergoing OPCABG demonstrated that maintaining MAP above 65 mmHg was associated with a reduced risk of AKI and shorter LOS. These finding highlight the importance of maintaining adequate intraoperative blood pressure to improve clinical outcomes in this patient population. A prospective research study is warranted to validate and refine our observations in the next step.

## Data Availability

Data will be available upon request from the corresponding author.

## Abbreviations

OPCABG: Off-pump coronary artery bypass grafting
AKI: Acute kidney injury
IOH: Intraoperative hypotension
MAP: Average arterial pressure
ICU: Intensive care unit
LOS: Length of stay
MI: Myocardial infarction
EPR: Electronic Patient Record
BMI: Body mass index
SII: Systemic immune-inflammation index
sCr: Serum creatinine
LVEF: Left ventricular ejection fraction
PCI: Percutaneous coronary intervention
CKD: Chronic kidney disease
DRG: Diagnosis-Related Group
